# Quantification of Persistence of *Escherichia coli* O157:H7 in Contrasting Soils

**DOI:** 10.1155/2011/421379

**Published:** 2010-09-08

**Authors:** A. Mark Ibekwe, Sharon K. Papiernik, Catherine M. Grieve, Ching-Hong Yang

**Affiliations:** ^1^USDA-ARS, U.S. Salinity Laboratory, 450 W-Big springs Roael, Riverside, CA 92507, USA; ^2^USDA-ARS, North Central Soil Conservation Research Laboratory, Morris, MN 56267, USA; ^3^Department of Biological Sciences, University of Wisconsin-Milwaukee, WI 53211, USA

## Abstract

Persistence of *Escherichia coli (E. coli)* O157:H7 in the environment is a major concern to vegetable and fruit growers where farms and livestock production are in close proximity. The objectives were to determine the effects of preplant fumigation treatment on the survival of *E. coli* O157:H7 in two soils and the effects of indigenous bacterial populations on the survival of this pathogen. Real-time PCR and plate counts were used to quantify the survival of *E. coli* O157:H7 in two contrasting soils after fumigation with methyl bromide (MeBr) and methyl iodide (MeI). Ten days after fumigation, *E. coli* O157:H7 counts were significantly lower (*P* = .0001) in fumigated soils than in the non-fumigated. Direct comparison between MeBr and MeI within each soil indicated that these two fumigants showed similar impacts on *E. coli* O157:H7 survival. Microbial species diversity as determined by DGGE was significantly higher in clay soil than sandy soil and this resulted in higher initial decline in population in clay soil than in sandy soil. This study shows that if soil is contaminated with *E. coli* O157:H7, fumigation alone may not eliminate the pathogen, but may cause decrease in microbial diversity which may enhance the survival of the pathogen.

## 1. Introduction

Appropriate management of farm waste such as manure is critical in controlling the spread of pathogens such as *E. coli* O157:H7 to vegetable crops. In most management schemes, fumigants are used for the control of plant pathogens, nematodes, and weeds before high-value cash crops such as strawberry and tomato are planted. Outbreaks of *E. coli *O157:H7 infections historically have been associated with consumption of undercooked ground beef; however, many recent outbreaks have resulted from consumption of contaminated raw vegetables, including lettuce [1–3]. Although many pathogens have been associated with fresh produce, *E. coli *O157:H7 is of particular concern because ingestion of relatively few cells can cause illness [4]*. E. coli* O157:H7 can survive for 60 to 120 days in water and in soil, and under dry and acidic conditions [5]. 

The steps in the production chain that have the greatest potential for pathogen contamination are soil preparation (use of uncomposted manures) and planting and growing (use of contaminated irrigation water and animal manures and manure from animals grazing locally or nearby) [6–8]. Prevention of preharvest contamination of fresh produce is an essential part of systems approach focused on interventions designed to achieve delivery of microbiological safe produce to consumers [9]. Suppression of human pathogens in agricultural soils and the subsequent prevention of spread into the food chain by contamination of produce must be realized by the adoption of best management practices. 

In the absence of known phytopathogens, many crops have exhibited an increased growth response when planted into soil that had been fumigated with MeBr [10] at the recommended application rate. One of the likely reasons for this observation may be that fumigation altered the microbial composition of the soil, either enhancing beneficial colonizers or reducing populations of deleterious rhizosphere colonizers. Fumigation is to control plant pathogens such as nematodes, soil-borne diseases, and weeds. The immediate impact of fumigation may be the reduction of certain bacterial species in the soil and the development of new communities after a few weeks [11, 12]. Some fumigants may be toxic to some microbes, and this may enhance the selection of microbes that may be beneficial to plants [11, 12]. Due to the increased focus on food safety related to fresh produce, there are more studies using real-time PCR to quantify pathogens such as *E. coli* O157:H7 in the environment [13, 14]. The main objectives of this study were to determine the effects of soil microbial diversity and fumigation on the survival of *E. coli* O157:H7 in soils contaminated with the pathogen. To accomplish these objectives, a preliminary study was conducted to determine the survival of the pathogen in both autoclaved and nonautoclaved soils at different concentrations of the pathogen. For our main objectives, both plate count and real-time PCR approaches were used to determine the survival of *E. coli* O157:H7 in the two soils. 

## 2. Materials and Methods

### 2.1. Bacterial Strain and Growth Conditions

*E. coli* O157:H7 (pGFP) strain 72 was kindly provided by Dr. Pina Fratamico of USDA-ARS [15]. This strain produces Shiga-like toxin *Stx*1 and *Stx*2 and the pGFP expressing green fluorescent protein (GFP) and ampicilin resistance. *E. coli* O157:H7 was cultured at 37°C overnight in modified Tryptic Soy broth (mTSB)(Difco Laboratories Inc., Cockeysville, MD) supplemented with 100 *μ*g of ampicillin ml^−1^ (Sigma, St Louis, MO). Cells were harvested by centrifugation at 3500 X g for 10 min and resuspended in phosphate buffered saline (PBS) (Fisher Scientific, Pittsburgh, PA) to a concentration of ~10^8^ CFU ml^−1^. Bacterial strains (except strain 72) used to determine the specificity and sensitivity of PCR assays were obtained from the National Animal Disease Center (Ames, IA) and were cultured on Luria-Bertani (LB) broth agar and Sorbitol MacConkey (SMAC) agar plates at 37°C.

### 2.2. Effect of Inoculum Density on the Survival of *E. coli* O157:H7 in Soils

Clay soil (willows silty clay, saline-alkaline) and sandy soil (dello loamy sand) were collected from Mystic Lake dry bed and the Santa Ana River bed, respectively, and treated as described by Ibekwe and Grieve [16]. The clay soil had a bulk density of 1.51 Mg m^−3^ with 3.7% sand, 49.1% silt, and 47.2% clay. The sandy soil has a bulk density of 1.67 Mg m^−3^ with 99.1% sand, 0.20% silt, and 0.70% clay. The moisture content of the clay soil was 4.02 % and that of sandy soil was 5.32%, before both were increased to about 12% at the start of the experiment. The pH of sand was 6.85 and that of clay was 7.45. Each soil (100 g dry wt) was placed in 150 ml beakers (18 beakers of each soil type) and the soil was autoclaved for 1 h, cooled for 24 h, and autoclaved again before use for the study. Eighteen beakers of each soil type were also kept unautoclaved for comparison. Inoculums were made in PBS and added to the appropriate soil beaker by spraying and mixing 10 ml of the culture mixture with a spray bottle (Sprayco, Detroit, Mich.; sanitized by soaking in 70% ethanol) on the surface of 100 g of soil to obtain the following inoculums concentrations in triplicate: 10^1^, 10^3^, 10^4^, 10^6^, 10^8^, and 0 for both autoclaved and unautoclaved soils.Serial dilution was made with 10 g portion of each soil for the enumeration of bacteria. Both the clay and sandy soils were treated the same. Soils were mixed for 5 min with sterile specula to homogeneously distribute the *E. coli* O157:H7, and covered with foil and incubated at 20° C in the growth chamber for the duration of the experiment. *E. coli* O157:H7 population was determined by plating on Tryptic soy agar (TSA; Becton Dickinson) plates containing 100 *μ*g of ampicillin ml^−1^ (TSA-A) at days 0 (inoculation), 1, 3, 5, 10, 20, 30, 40, 50, and 60. The GFP-labeled *E. coli* O157:H7 colonies were counted under an UV light.

### 2.3. Growth Chamber Experiment after Fumigation

The fumigant methyl iodide (MeI, iodomethane, >99% purity) was purchased from Chem Service (West Chester, PA) and methyl bromide (MeBr >99% purity) was obtained from Great Lakes Chemical Company (West Lafayette, IN). Plastic trays (58.2 × 43.2 × 18.5 cm) were filled with approximately 40 kg of soil. The soils were irrigated with approximately 2.2 ×10^8^ *E. coli* O157:H7. Bacteria were inoculated into the irrigation lines with a Cole-Parmer HPLC pump (Cole-Parmer, Chicago, Illinois) and delivered through PVC pipes to each tray with five surface drip lines. The five drip lines delivered about 10^6^ CFU g^−1^ *E. coli* O157:H7 at time zero. Soil samples were collected on the day of inoculation for community analysis, *E. coli* O157:H7(pGFP) concentration and heterotrophic plate counts. After the initial sample collection, trays were manually covered with a virtually impermeable plastic film; 0.038 mm Hytibar film (Klerk Plastics, Belgium) and fumigants were applied. 

Fumigant rates and application methods were selected according to the recommended field application rate for each chemical by California Department of Pesticide Regulation ( http://www.cdpr.ca.gov/). To avoid the emission of fumigants to the growth chamber, syringes were used to inject fumigant (MeBr-gas and MeI-liquid) into the trays; injection ports covered immediately with duct tape and left in the growth chamber for 10 days. After 10 days, trays were moved outside and the Hytibar film was removed. Trays remained outside in an area covered with barb wires, opened and aerated for 2 days before they were moved back to the growth chamber for the continuation of the experiment. At this point, a total of 14 days has elapsed since fumigation, and soil samples were collected for *E. coli* O157:H7(pGFP) concentration, bacterial diversity, and heterotrophic plate counts. 

Soils were irrigated with 50% Hoagland solution [17] in the two growth chambers. The experiment was a completely randomized design with two replications. The clay soil was irrigated with distilled water daily and received nutrient solution weekly. The sandy soil received the above nutrient solution twice daily, because this was a very poor river bank soil with poor nutrients for plant growth. Soil samples were collected weekly for 5 weeks for *E. coli* O157:H7, heterotrophic plate counts and for total bacterial DNA extraction. The samples were collected in separate sterile petri dishes or collection bags. Negative controls were collected first with sterile spatula. Soil was transferred to ziplock bags and 10 g sample was used for serial dilution. *E. coli*/pGFP colonies were enumerated under a hand-held Spectroline ultraviolet lamp (Spectronics Corporation, Westbury, N.Y).

### 2.4. DNA Extraction, PCR Amplification, and DGGE Analysis

Community DNA was extracted from 0.5 g soil with the Ultra Clean Soil DNA Kit (MoBio Laboratories, Solana Beach, CA) according to the manufacturer's protocol and stored at −20° C. A 236-bp DNA fragment in the V3 region of the small subunit ribosomal RNA genes of eubacteria was amplified by using primer set PRBA338f and PRUN518r [18]. Ready-To-Go PCR beads (Amersham Pharmacia Biotech, Piscataway, NJ) and 5 pmol of primers in a total volume of 25 ml were used in the PCR reaction. PCR amplifications were done under the following conditions: 92°C for 2 min; 30 cycles of 92°C for 1 min, 55°C for 30 s, 77°C for 1 min followed by a final extension at 72°C for 6 min. DGGE was performed with 8% (wt/vol) acrylamide gels containing a linear chemical gradient ranging from 30% to 70% denaturant with 100% defined as 7 M urea and 40% formamide. Gels were run for 3 h at 200 V with the Dcode Universal Mutation System (Bio-Rad Laboratories, Hercules, CA). DNA was visualized after ethidium bromide staining by UV transillumination and photographed with a Polaroid camera. DNA fingerprints obtained from the 16S rRNA banding patterns on the DGGE gels were photographed and digitized using ImageMaster Labscan (Amersham-Pharmacia Biotech, Uppsala, Sweden) and analyzed [11]. The comparison of diversity was done by using a one-way analysis of variance, and Tukey HSD test for post hoc analysis [19]. Diversity was calculated by using the Shannon index of diversity (*H*) to compare changes in diversity of microbial communities within all treatments at each time [20] by using the following function:

(1) $$H\;=\;-\;\sum P_{i}\;\log\;P_{i},$$

when *P*_*i*_ = *n*_*i*_/*N*, *n*_*i*_ is the height of peak, and *N* is the sum of all peak heights in the curve.

### 2.5. Primer and Probe Design for Real-Time PCR. 

Genomic DNA Was Isolated from pure culture of *E. coli* O157:H7, grown for 12 h at 37°C and extracted with the Qiagen tissue kit (QIAamp DNA Mini Kit; Valencia, CA). DNA extracted from O157:H7 was used for the construction of standard curve and for the determination of detection limits of the *E. coli* by real-time PCR. Total bacterial DNA was extracted from soil with the Ultra Clean Soil DNA Kit (MoBio Laboratories, Solana Beach, CA) as stated above and stored at −20°C. Primers and probes used for the detection and quantification of the *stx*1, *stx*2, and the *eae* gene in *E. coli* O157:H7 were as described [21, 22]. Real-time, quantitative PCR was performed with the iCycler iQ (Bio-Rad, Hercules, CA) as described by Ibekwe et al. [21]. Briefly, PCR was performed in a total volume of 50 *μ*l volume containing 200 *μ*M of dNTPs, 2 *μ*l of genomic DNA from each concentration, 2.5 U of AmpliTaq Gold polymerase, 5 *μ*l of 10X TaqMan buffer (PE Applied Biosystems, Foster City, CA), 0.3 *μ*M of each primer, 0.1 *μ*M of probe, and 3.5 mM of MgCl_2_. Genomic DNA purified from *E. coli* O157:H7 was used as a template for the positive control and no template for negative control. PCR was performed using the following cycle conditions: denaturation at 95°C for 10 min, 50 cycles of 94°C for 20 s, 55°C for 30 s, 72°C for 40 s, followed by a 5 min extension at 72°C and a hold at 4°C. Standard curves generated from plotting the threshold cycle (*C*_*T*_) versus log_10_ of starting DNA quantities (pg) were used for determining the detection limit of the assay. Optimization of the multiplex assay was done as previously discussed [21, 22]. Amplification efficiency (E) was estimated by using the slope of the standard curve and the formula: E = (10^−1/slope^)−1. Reaction with 100% efficiency generated a slope of –3.32.

### 2.6. Data Analysis

*E. coli* O157:H7 concentrations were converted to log CFU g^−1^ for regression analysis. Statistical analyses were done with the general linear model (GLM) procedure of the Statistical Analysis System [19]. The population data were log transformed to obtain a normal distribution of the data. Comparisons between pairs of treatment means at any date were accomplished with the Tukey's test. The log-transform data of *E. coli* O157:H7 population size of all individual samples were plotted over time after inoculation, and analyzed by regression analysis [19]. Plate counts and real time PCR data were transformed to Log_10_ values and survival curves were obtained by plotting the logarithm of survivors against the treatment time. The survival data were fitted to a biphasic model as proposed by Coroller et al. [23, 24] with the Geeraerd and Van Impe inactivation model-fitting tool (GInaFiT) as shown in (2) and (3) and as described by Franz et al. [25]:

(2) $$\begin{array}{c} N\left(t\right)=\frac{N_{0}}{1+10^{\alpha }}\left[10^{-{\left(t/\delta_{1}\right)}^{p}+\alpha }+10^{-{\left(t/\delta_{2}\right)}^{p}}\right], \end{array}$$

(3) $$\alpha ={\log\;}_{10}\;\left(\frac{f}{1-f}\right),$$

where *N* is the number of survivors, *N*_0_ is the inoculums size; *t* is the time; *p* is the shape parameter, when *p* > 1 a convex curve is observed; when *p* < 1 a concave curve is observed, when *p* = 1 a linear curve is observed. The scale parameter, *δ*, represents the time needed for first decimal reduction. *f*, varying from 0 to 1, is the fraction of subpopulation 1 in the population. Another parameter, *α*, varying from negative infinity to positive infinity, is obtained by logit transformation of *f* as shown in equation 2. The strong correlation between the scale (*δ*) and the shape (*p*) parameters makes it possible for the double Weibull model to fit most of the shapes of deactivation curves. Additionally, when *δ*_1_ = *δ*_2_, the double Weibull model can be simplified into a single Weibull model, and the survival curve can be described by only three parameters. A very important and useful parameter, time to detection limit (Td) can also be calculated when using GInaFiT to fit the experimental survival data.

## 3. Results

### 3.1. *E. coli* O157:H7 Survival in Clay and Sandy Soils

**Figure 1:**
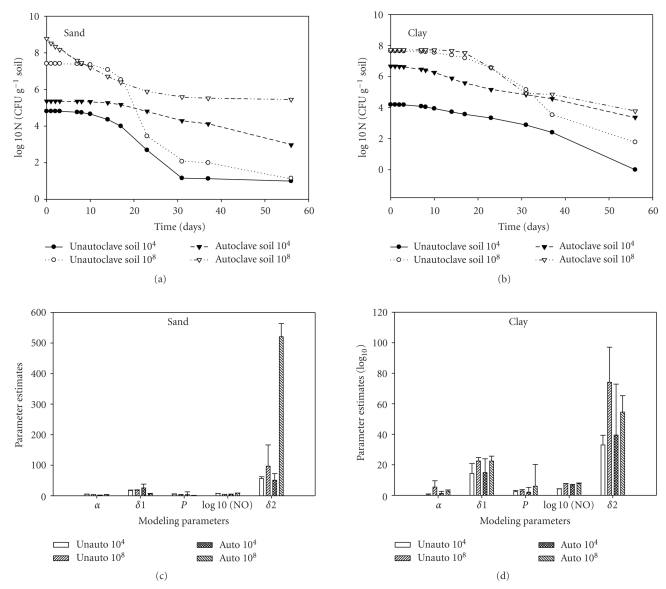
Survival of *E. coli* O157:H7 in autoclaved and unautoclaved (a) sand and (b) clay soils after 60 days incubation in a growth chamber at 20°C. *E. coli* O157: H7 was enumerated on TSA-ampicilin plates from soil with different concentrations of pathogen. Each point represents the average of triplicate sampling, and the bars at each point indicate survival coefficient obtained at each sampling point. All were log transformed as reported in the text. Double Weibull model parameters of *E. coli* O157:H7 in autoclave and nonautoclave sandy and clay soils (c, d)

The effects of inoculum density on the survival of *E. coli* O157:H7 in sandy and clay soils was first determined in the two soils used for this study. This was done to determine the influence of indigenous microorganism on the survival of *E. coli* O157:H7 in autoclaved and unautoclaved soils. Data from the survival study showed that within the first 7 days *E. coli* O157:H7 populations decreased by ca. 0.24 log_10_CFU g^−1^ in the 10^4^ dilution and by 0.67 log_10_CFU g^−1^ in the 10^8^ dilution for the unautoclaved soil (Figures 1(a) and 1(b)). The reverse was the case with autoclaved soil where there was an increased in population by 2.13 log_10_ CFU g^−1^ in the 10^4^ dilution and an increased of 1.68 log_10_ CFU g^−1^ in the 10^8^ dilution. Survival curves showed a concave curvature in autoclave sandy soil and a convex curvature in the unautoclaved sandy soil (Figure 1(a)). In the clay soil, the shape parameter was different from sandy soil with soils with inoculums density of 10^8^ showing the convex shape whereas soils with cells at 10^4^ showed either concave or linear shape. Modeling parameters (alpha (**α*)*, delta (**δ*)*, and the shape parameter-*p*) were calculated from equation (2) and (3) used to explain the inactivation kinetics. More variations in **δ** values were observed from different soils (Figures 1(c) and 1(d)). When the strain was characterized in sandy and clay soils, distinct *δ*_1_ and *δ*_2_ were observed indicating that the two subpopulations behaved differently in both soils, thus the survival data in both soils might not be simplified into the single Weibull model that can be described by only three parameters, *α*, **δ** and *p*. The initial sharp decrease in cell numbers in sandy soil (concave shape) might largely be attributed to the faster decline of subpopulation as shown with smaller *δ*_1_ (Figure 1(c)). However, with the time going, the subpopulation with greater *δ*_2_ (i.e., the more resistant) dominated the cell population, leading to a slower and steadily decline of the cell concentration as the curves showed little or no decline. After the first 10 days, *E. coli* O157:H7 populations in unautoclaved soil declined considerably more rapidly than in autoclaved soil below the detection limits of 10^2^ CFUg^−1^ soil. After 60 days, the concentration of *E. coli* O157:H7 in the 10^4^ dilution was undetectable by plate count, and there was a 6.18 log_10_CFU g^−1^ reduction for the 10^8^ dilution. Survival of pathogen was greater in the sandy soil (*P* = .05) than clay unautoclaved soil within the first 7 days (Figure 1). *E. coli* O157:H7 at 10^8^ CFU g^−1^ dilutions survived for more than 60 days in both unautoclaved and autoclaved soils used in this study.

### 3.2. Impact of Fumigants on Survival *E. coli* O157:H7 in the Growth Chamber

Before the enumeration of *E. coli *O157:H7 in the different matrices, background concentrations of heterotrophic bacterial was determined. The initial heterotrophic plate count in soil was 2.1 × 10^8^ CFU g^−1^. After storage at 20°C in the growth chamber, the total aerobic plate counts decreased steadily from ca. 10^8^ to ca. 10^6^ CFU g^−1^ ± 10^2^ during the experimental period in both soils. There were no differences in the levels of heterotrophic plate count in the two soils during the study period (data not shown). Mean comparison by days and methods were used to determine the impact of fumigants on the survival of *E. coli *O157:H7 in the two soils after fumigation (Table 1). Since one of our objectives was to determine the effects of fumigants on *E. coli* O157:H7 on a weekly basis, direct comparison of the two fumigants and the control was done using plate count and real-time PCR to quantify the concentrations of *E. coli* O157:H7. In the growth chamber soil, ten days after fumigation, *E. coli* O157:H7 was significantly lower (*P* = .0001) in fumigated soils than the control clay soil at the recommended application rate. During the rest of the study, there were no significant differences on the effect of the two fumigants on the pathogen, except on day 36 (*P* = .046) where the effects varied. Real-time PCR analysis showed that 10 days after fumigation, *E. coli* O157:H7 concentration in non-fumigated soils was significantly higher (*P* = .002) in sandy soil than clay soil. There were no significant differences (*P* = .56) in pathogen concentration during day 23 when real-time PCR was used for the analysis. The same effect was observed during day 36 and 50 (data not shown). Direct comparison between MeBr and MeI within each soil showed that neither had significant greater impact on *E. coli* O157:H7. 

**Figure 2:**
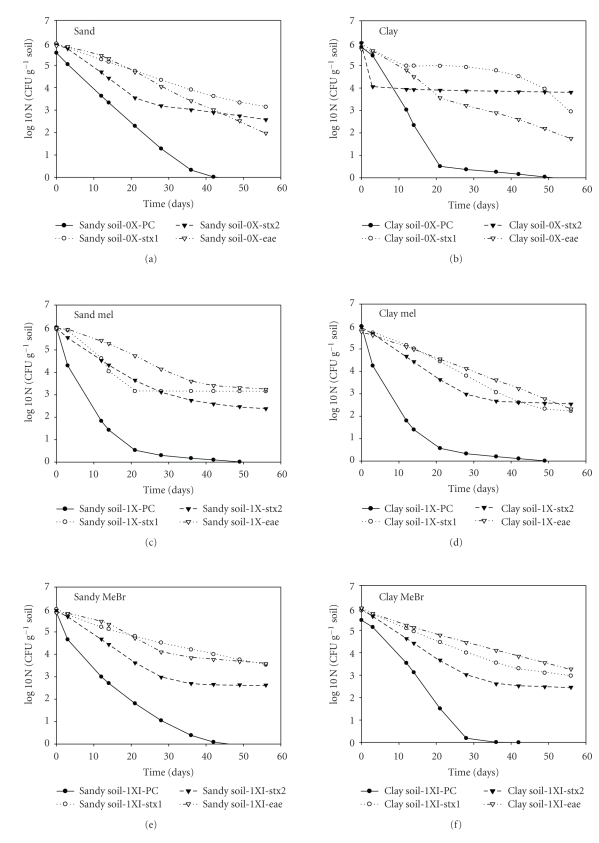
Quantification of * E. coli* O157:H7 in growth chamber after 60 days in fumigated soils. Fumigant (MeBr) was applied at the normal application rate: non fumigated control: (a) (sandy soil), (b) (clay soil); normal application rate for MeI (c) (sandy soil), (d) (clay soil); normal application rate for Mebr (e) (sandy soil), D (clay soil); *E. coli* O157:H7 were enumerated from sandy and clay soil by plate count and by real-time PCR (RT-PCR) using *stx*1, *stx*2, and the *eae* genes.

The majority of the survival curves (Figure 2) showed a concave shape, with a relatively fast initial decline followed by a slower decline phase. Survival reached the detection limit faster in clay soil than in sandy soil without fumigation using plate counts (Figures 2(a) and 2(b)). When the pathogen was exposed to fumigants (MeI), inactivation was faster than in control, especially with plate count (Figures 2(c) and 2(d)). The same pattern was observed with MeBr (Figures 2(e) and 2(f)). However, for both control treatments the population size did not reach the detection limit (ttd) of 10^2^ CFU g^−1^ during the experiment due to earlier onset of tailing at about 35 days using real-time PCR. Also, both soils showed that it took less than a day to inactivate the first log_10_ of microbial population in most of the fumigated samples. 

**Figure 3:**
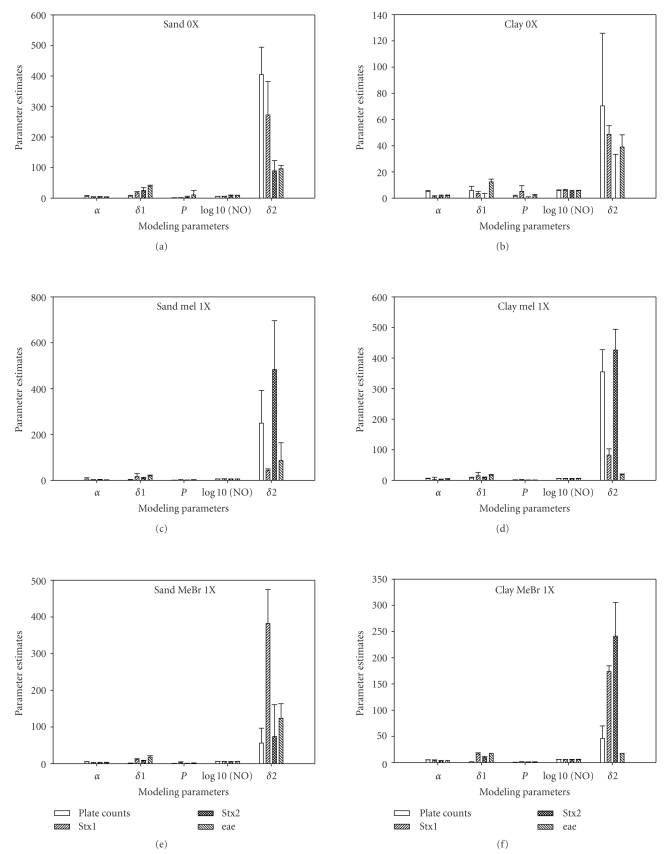
Double Weibull Model parameters of *E. coli* O157:H7 in nonfumigated sandy and clay soils (a and b), fumigated with MeI in sandy and clay soils (c and d), and fumigated with MeBr in sandy and clay soils (e and f).

Effects of soil types on the survival *E. coli* O157:H7 in clay and sandy soils after fumigation was model by fitting the experimental data into the survival functions (Figure 3). Similar modeling parameters (**α**, **δ**, and *p*) were calculated when they were inoculated into the same soil (Figures 3(a)–3(f)). However, there more little variations in these parameters from the two soils, except the **δ** values. When the pathogen was characterized in sandy and clay soils, distinct *δ*_1_ and *δ*_2_ were observed indicating that the two subpopulations behave differently in both soils. The goodness-of-fit statistics (*R* ^2^) did not differ significantly between survival curves in sandy soil irrespective of fumigants or no fumigant. The same effect was observed in clay soil, indicating that the model is suitable to fit survival curves of *E. coli* O157:H7 in an array of different soils.

### 3.3. Influence of Microbial Diversity on *E. coli* O157:H7 Survival

**Figure 4:**
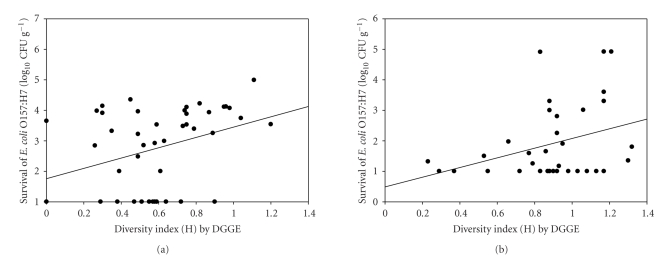
Obseved points (●) and relations (solid lines between survival and microbial diversity in soil. (a) sandy soil-growth chamber; (b) clay soil- growth chamber.

DGGE analysis of 16S rRNA fragments was used to examine the effects of MeBr and MeI on soil microbial communities during week 1 to7 after fumigation. The most drastic effect occurred on the first week of the experiment where there was a significant effect (*P* ≤ .05) of fumigants as determined by the Shannon-Weaver index of diversity between clay and sandy soil (Table 2). During this period, microbial diversity in the MeBr fumigated treatments was significantly lower (*P* = .0003) in sandy soil than in clay soil at the recommended application rate. The same effect was observed with MeI fumigated soil. Bacterial communities were not different at week three (*P* = .13), week four (*P* = .06), and week five (*P* = .11). However, at week seven there was a significant (*P* = .0001) shift in microbial community structure as determined by diversity index with all the treatments (Table 2), and the effect was greater in sandy soil than clay soil. This resulted in the initial higher survival rate of *E. coli *O157:H7 in sandy soil compared to clay soil. Microbial diversity (expressed as Shannon Weaver index of diversity *H*) was positively correlated with survival of *E. coli* O157:H7 in sandy soils (Figure 4(a); *r* ^2^ = 0.56, *P* = .015) and in clay soil using the plate count method (Figure 4b; *r* ^2^ = 0.47; *P* = .019). Using data from real-time PCR analysis, survival of *E. coli *O157:H7 were positively correlated with microbial diversity in both clay and sandy soils (data not shown).

## 4. Discussion

Before the start of this experiment, a preliminary study was conducted in autoclaved and unautoclaved soil to determine the influence of indigenous soil microorganisms on the survival and growth of *E. coli* O157:H7 (Figures 1(a) and 1(b)). The antagonistic effect of indigenous soil microorganisms was likely a factor in killing *E. coli *O157:H7 cells in unautoclaved soils used in this study. *E. coli* O157:H7 was inactivated more rapidly in unautoclaved soil than in autoclaved soil in all the different dilutions of* E. coli* O157:H7 used as inoculums (Figures 1(a) and 1(b) for 10^4^ and 10^8^ CFU g^−1^). Our study is in agreement with Jiang et al. [26] who showed that small numbers of *E. coli* O157:H7 from an unautoclaved soil were only detected by enrichment culture, and survived for longer period of time at 15°C than 21°C. This suggests that there was a small population of cells that have survived in the soil under different environmental stress. The long-term survival of this pathogen in the environment has been reported by many authors [6, 27–31], but very little has been done on the survival in fumigated soil. We have shown from this study that *E. coli* O157:H7 can survive in fumigated soils for over 60 days due to long-term persistence of a small percent of the population.

Our study showed that *E. coli* O157:H7 can survive longer in sandy soil than in clay soil during a short term experiment. However, populations persisted longer in clay than in sandy soil during a long-term study. Our results showed that *E. coli *O157:H7 survived longer than 60 days in both soils. Others have reported survival of more than 54 days in manure amended soil [28, 32–34] and 34 days or more in sandy loam soil amended with cow manure [26, 35], and over 90 days in clay soils (8, 40). Others reported longer *E. coli *O157:H7 survival times of between 154 and 217 d in soils amended with inoculated compost [27]. This study with longer survival period agrees with our study because both studies relied on inoculating the substrate with relatively high densities of the pathogen (>10^6^  CFU g^−1^). Also, during our preliminary experiment with *E. coli* O157:H7 with population of lesser than 10^4^  CFU g^−1^, survival of the pathogen was less than seven days. This is in agreement with Franz et al. [36] that monitored the fate of the pathogens in manure-amended soil after they declined to below detection limit within a short period with low and more realistic levels of pathogens inoculated into manure (approximately 10^2^  CFU g^−1^). Therefore, the survival of *E. coli* O157:H7 in the environment may depend on the initial concentration at the beginning of the experiment. 

In this study, we used the double Weibull model, which is the cumulative form of the underlying distribution of individual inactivation kinetics, and it was a suitable model for describing the decline of *E. coli* O157:H7. The survival curves generated from our study in most cases showed a convex fitting, indicating changes in biological stress over time. The model is sufficiently flexible to account for different survival patterns and has been previously used to model thermal inactivation of *Listeria monocytogenes* in sucrose solutions of various water activities [37] and the survival of* E. coli* O157:H7 in manure amended soil [32]. Franz et al. [32]; Van Boekel, [38]; Peleg, [39] discussed the different processes and mechnisms responsible for the different shape parameters during inactivation of *E. coli* O157:H7 in soil. They pointed out that even though the Weibull model is an empirical model, it can be linked to physiological properties at population level and that the population is heterogeneous with respect to the stress encountered in the soil. These authors noted that a convex curve would mean that the remaining cells become increasingly susceptible to stress, and the cells are therefore subjected to more damages with time. A linear survival curve means that inactivation does not depend on time or other biological activities and the concave survival curve means that sensitive members of the population are rapidly eliminated and that the sturdier survivors remain. 

Longer persistence of *E. coli* O157:H7 in clay soil may be influenced by interaction between soil particles in the clay particle sizes that provided niches for the pathogen and moisture/nutrients within the niches. Other factors that contributed to the survival of pathogen in soil were soil microbial diversity. Recently, the effects of *E. coli* O157:H7 was assessed in a loamy sand soil obtained from species-rich grassland, in which the microbial community composition had been modified by progressively enhanced fumigation depths [40]. The authors showed that *E. coli* O157:H7 in the soils with modified community structures due to fumigation was clearly consistent with the hypothesis that within the single selected habitat (soil), which was relatively unaffected with respect to abiotic conditions like pH, moisture and soil chemical conditions, microbial community structure was the main determinant of the survival of the pathogen. With the present study we found that the values of the log reduction time and the shape parameter of the double Weibull model were higher for clay soils compared to sandy soils. This means that with sandy soils the initial rate of decline of *E. coli* O157:H7 was faster than in clay soil. *E. coli* O157:H7 was more vulnerable to mortality during the first few weeks in the sandy soil than in clay soil. Finer-textured (clayey) soils result in prolonged survival of introduced bacteria compared with coarser-textured (sandy) soils because of higher availability of protective pore spaces against feeding by soil fauna like protozoa [41]. This could explain the faster initial decrease in *E. coli* O157:H7 numbers in the sandy soils compared with the loamy soils, but survivors are increasingly more sturdy compared with survival in the clay soils. The implication of long-term survival of this pathogen in the environment may involve the recontamination of the environment after the initial contamination event from few surviving strains.

*E. coli* O157:H7 was significantly lower in fumigated soils than the control at the normal application rate during the first 2 weeks of the experiment [42]. However, survival was significantly affected by soil type, with survival greater in sandy soil in the short run than clay soil [43]. However, long-term persistence occurred in clay soil than sandy soil due to properties of clay soil. None of the fumigants showed significant higher toxicity effects on the pathogen at the normal application on the long run, and toxicity was higher in fumigated soil than nonfumigated soils during the first two weeks of the study. Therefore, MeBr and MeI have identical toxicity effects on *E. coli* O157:H7 at the normal application rate.

## Figures and Tables

**Table 1:** Mean comparison by days and methods (log_10_ *g* ^−1^) of survival of *E. coli* in soil after fumigation.

TRT	Day 10-PC	Day 23-PC	Day 28-PC	Day 36-PC	Day 50-PC	Day 10-Stx 1	Day 10-Stx 2	Day 10-eae	Day 23-Stx 1	Day 23-Stx 2	Day 23-eae	Day 28-Stx 1	Day 28-Stx 2	Day 28-eae
Sb1/2e	4.22 ab	4.11 a	3.25 a	2.48 a	1.00 a	6.12 ab	5.67 a	7.10 abc	4.71 a	3.34 a	6.12 a	4.81 ab	2.86 ab	5.97 ab
sb1e	3.91 ab	4.07 a	3.22 a	2.00 ab	1.00 a	5.72 ab	4.72 ab	6.77 abc	4.21 a	2.57 a	6.00 a	7.11 a	3.90 ab	5.41 ab
sI1/2e	3.39 abc	3.74 a	1.00 a	1.00 b	1.00 a	6.73 a	5.57 a	7.87 a	5.73 a	3.88 a	6.51 a	4.95 ab	2.85 ab	5.88 ab
sI1e	3.88 ab	3.93 a	2.92 a	1.00 b	1.00 a	6.09 ab	4.19 abc	7.09 abc	5.43 a	3.73 a	6.66 a	5.65 ab	3.36 ab	6.81 a
so0e	4.99 a	3.48 a	4.16 a	2.84 a	3.53 a	6.34 a	4.75 ab	7.41 ab	5.07 a	3.17 a	4.37 a	4.42 ab	4.16 ab	5.29 ab
cb1/2e	3.65 ab	3.96 a	3.21 a	1.00 b	2.18 a	4.09 bc	1.00 e	3.59 bc	4.59 a	2.88 a	3.55 a	3.90 b	1.00 b	2.44 b
cb1e	3.67 ab	2.25 a	2.17 a	1.00 b	1.00 a	5.09 abc	3.10 cd	5.98 abc	4.48 a	3.57 a	4.47 a	4.45 ab	3.41 ab	2.44 b
cI1/2e	1.00 c	1.99 a	1.25 a	1.00 b	1.00 a	4.67 abc	2.45 d	4.30 abc	4.39 a	2.44 a	3.79 a	3.61 b	2.60 ab	3.19 b
cI1e	4.16 ab	4.44 a	0.58 a	1.00 b	1.00 a	5.34 abc	3.98 bc	6.65 abc	4.78 a	3.36 a	4.75 a	4.16 b	1.22 ab	5.02 ab
co0e	1.41 bc	3.85 a	3.01 a	2.59 a	3.10 a	3.27 c	2.44 d	3.08 c	5.32 a	2.57 a	6.24 a	4.10 b	5.86 a	3.50 ab

Means with different letters within each column are significantly different at *P* ≤ .05 using Tukey's Studentized Range Test. s = sandy soil; b = methyl bromide, I = methyl Iodide; c = clay soil; 1/2 = 50% recommended application rate; 1 = recommended application rate; e = sample were inoculated with *E. coli *O157:H7.

**Table 2:** Numerical analysis of DGGE bands from growth chamber soil samples with Shannon index of diversity (*H*).

Treatment	Week 1	Week 3	Week 4	Week 5	Week 7
CB 0.5X+E*	1.34 abc	0.88 a	1.03 a	0.13 a	0.58 abc
CB 0.5X−*E*	0.88abcd	0.66 a	0.30 a	0.53 a	0.91 a
CB 1.0X+*E*	1.56 a	0.55 a	0.65 a	0.37 a	0.70 ab
CB 1.0X−*E*	1.27 abc	0.33 a	0.82 a	0.71 a	0.30 bc
CI 0.5X +*E *	1.12 abc	0.95 a	0.85 a	0.38 a	0.70 ab
CI 0.5X −*E*	1.22 abc	0.72 a	0.94 a	0.86 a	0.96 a
CI 1.0X+*E *	1.48ab	0.68 a	0.79 a	0.67 a	0.78 ab
CI 1.0X−*E*	1.45 ab	0.57 a	0.42 a	0.89 a	0.79 ab
CO 0+*E*	1.10 abcd	0.76 a	0.89 a	0.78 a	0.82 ab
SB 0.5X+*E*	0.82 abcd	0.96 a	0.49 a	0.39 a	0.001 c
SB 0.5X−*E*	0.45 bcd	0.98 a	0.35 a	0.47 a	0.58 abc
SB 1.0X+*E*	0.30 cd	0.75 a	0.55 a	0.51 a	0.001 c
SB 1.0X−*E*	0.27 cd	1.04 a	0.57 a	0.29 a	0.64 ab
SI 0.5X +*E*	0.79 abcd	1.20 a	0.76 a	0.90 a	0.59 ab
SI 0.5X −*E*	0.49 abcd	0.74 a	0.63 a	0.26 a	0.30 bc
SI 1.0X+*E*	0.75 abcd	0.87 a	0.30 a	0.57 a	0.83 ab
SI 1.0X−*E*	0.001 d	0.73 a	0.49 a	0.72 a	0.45 abc
SO 0+*E*	1.11 abc	0.89 a	0.52 a	0.38 a	0.38 abc
SO 0−*E*	0.95abcd	0.75 a	0.61 a	0.59 a	0.59 ab

Means with different letters within each column are significantly different at *P* ≤ .05 using Tukey's Studentized Range Test. *C = clay soil; B = methyl bromide, I = methyl Iodide; S = sandy soil; 0.5X, and 1X represents half agricultural application rate and recommended agricultural application rate. + E = *E. coli* O157:H7 applied to the samples and – E not applied to the sample.
